# Coral reef mesopredators switch prey, shortening food chains, in response to habitat degradation

**DOI:** 10.1002/ece3.2805

**Published:** 2017-03-18

**Authors:** Tessa N. Hempson, Nicholas A. J. Graham, M. Aaron MacNeil, David H. Williamson, Geoffrey P. Jones, Glenn R. Almany

**Affiliations:** ^1^ ARC Centre of Excellence for Coral Reef StudiesJames Cook UniversityTownsvilleQldAustralia; ^2^ Lancaster Environment CentreLancaster UniversityLancasterUK; ^3^ Australian Institute of Marine ScienceTownsvilleQldAustralia; ^4^ Department of Mathematics and StatisticsDalhousie UniversityHalifaxNSCanada; ^5^ College of Marine and Environmental SciencesJames Cook UniversityTownsvilleQldAustralia; ^6^ CRIOBE–USR 3278CNRS–EPHE–UPVD and Laboratoire d'Excellence “CORAIL”Perpignan CedexFrance

**Keywords:** coral reefs, coral trout, food chains, habitat degradation, mesopredator, *Plectropomus maculatus*, prey switching, stable isotopes

## Abstract

Diet specificity is likely to be the key predictor of a predator's vulnerability to changing habitat and prey conditions. Understanding the degree to which predatory coral reef fishes adjust or maintain prey choice, in response to declines in coral cover and changes in prey availability, is critical for predicting how they may respond to reef habitat degradation. Here, we use stable isotope analyses to characterize the trophic structure of predator–prey interactions on coral reefs of the Keppel Island Group on the southern Great Barrier Reef, Australia. These reefs, previously typified by exceptionally high coral cover, have recently lost much of their coral cover due to coral bleaching and frequent inundation by sediment‐laden, freshwater flood plumes associated with increased rainfall patterns. Long‐term monitoring of these reefs demonstrates that, as coral cover declined, there has been a decrease in prey biomass, and a shift in dominant prey species from pelagic plankton‐feeding damselfishes to territorial benthic algal‐feeding damselfishes, resulting in differences in the principal carbon pathways in the food web. Using isotopes, we tested whether this changing prey availability could be detected in the diet of a mesopredator (coral grouper, *Plectropomus maculatus*). The δ^13^C signature in grouper tissue in the Keppel Islands shifted from a more pelagic to a more benthic signal, demonstrating a change in carbon sources aligning with the change in prey availability due to habitat degradation. Grouper with a more benthic carbon signature were also feeding at a lower trophic level, indicating a shortening in food chains. Further, we found a decline in the coral grouper population accompanying a decrease in total available prey biomass. Thus, while the ability to adapt diets could ameliorate the short‐term impacts of habitat degradation on mesopredators, long‐term effects may negatively impact mesopredator populations and alter the trophic structure of coral reef food webs.

## 1 Introduction

Coral reef ecosystems are rapidly being degraded through multiple disturbances from human activities and the cumulative impacts of climate change (Ban, Graham, & Connolly, 2014; Graham, Jennings, MacNeil, Mouillot, & Wilson, 2015; Hoegh‐Guldberg et al., 2007). Such loss of habitat is predicted to be one of the most important drivers of marine defaunation in the next century (McCauley et al., 2015). Habitat degradation directly threatens coral‐dependent species of coral reef fish, resulting in extensive changes in abundance and diversity within reef fish assemblages (e.g., Jones, McCormick, Srinivasan, & Eagle, 2004; Pratchett et al., 2008; Wilson, Graham, Pratchett, Jones, & Polunin, 2006; Wilson et al., 2008, 2010). Reef fishes differ in their response to habitat degradation depending on both the type of disturbance and the degree of specialization in resource requirements (Graham et al., 2011). Although reductions in live coral cover and habitat structural complexity often lead to declines in the abundances of many reef fishes, some species may increase, resulting in shifts in assemblage structure (Bellwood, Hoey, Ackerman, & Depczynski, 2006). For example, degraded reefs are typified by increases in algal cover that can benefit herbivorous fishes, at least in the short term (Pratchett et al., 2008). While there is a reasonable understanding of how coral reef fishes that are directly reliant on corals respond to reef habitat degradation (Wilson et al., 2006), relatively little is known about how indirect effects mediated via the food web affect higher trophic levels.

The long generation time of many larger predatory species at higher trophic levels means that the impacts of food web changes may take years or even decades to become apparent. This is one of the primary reasons cited by Estes et al. (2011) for the cryptic nature of “trophic downgrading,” a process whereby large consumers are being lost from ecosystems at a global scale. As high‐level consumers are widely considered to exert important top‐down effects in food webs (Duffy, 2003), trophic downgrading could have wide‐ranging implications for ecosystem structure and a broad suite of ecological processes (McCauley et al., 2010).

Medium‐ to large‐bodied reef fishes that are mesopredators (e.g., Serranidae, Lutjanidae, and Lethrinidae) are important for mediating energy flow between herbivores and apex predators on coral reefs (Polovina, 1984). Many mesopredators are also targeted by commercial, recreational, and subsistence fisheries (Cinner et al., 2009; Friedlander & DeMartini, 2002; GBRMPA, 2014; Lédée, Sutton, Tobin, & De Freitas, 2012). However, our understanding of the effects of habitat disturbance on these species is relatively poor. Changing prey availability is one of the primary mechanisms through which habitat disturbance can affect mesopredator trophic dynamics, and can be an important driver of total piscivore abundance (Graham et al., 2007; Wilson et al., 2008). While large mobile apex predators may have the capacity to respond to localized degradation by moving to remnant healthy and productive habitats, less mobile reef‐associated mesopredators may need to modify their prey selection in degraded habitats in order to fulfil their energetic requirements (Shpigel & Fishelson, 1989). Consequently, the persistence of reef‐associated mesopredator populations will depend to a large extent on their ability to adapt their diets as reef habitats become increasingly altered.

Gut content analysis has been traditionally used to collect dietary data in marine predators (Cortés, 1997). However, this technique has a number of limitations, including being only a snapshot view of a consumer's diet (Pinnegar & Polunin, 1999), loss of regurgitated prey during capture, unidentifiable stomach contents, and differential digestion rates among prey types (Baker, Buckland, & Sheaves, 2014). Stable isotope analyses of tissue samples are a powerful tool for understanding the trophic ecology of consumers (Boecklen, Yarnes, Cook, & James, 2011; Letourneur et al., 2013) that provide a dietary signal integrated over extended time periods (Phillips & Gregg, 2003; Pinnegar & Polunin, 2000). A consumers' δ^15^N isotopic signature is typically enriched relative to their food source, making it possible to calculate a predator's trophic position (TP; Layman, Arrington, Montaña, & Post, 2007; Post, 2002a; Post, Pace, & Hairston, 2000). In contrast, δ^13^C signatures remain relatively unchanged up the food web, providing a means to identify carbon sources (Fry, 2006; McMahon, Thorrold, Houghton, & Berumen, 2016; Peterson, 1999). In the marine environment, the primary sources of variation in predator δ^13^C signatures include geographic position (i.e., latitude or inshore vs. offshore production; McMahon et al., 2016), alternative carbon pathways (i.e., benthic vs. pelagic production; Hobson, Piatt, & Pitocchelli, 1994), and prey choice (Fry & Sherr, 1984).

Stable isotopes can also uniquely quantify changes in total food web structure (Post, 2002a). Food chain length (FCL) is a central concept in trophic ecology and a widely accepted metric used to describe changing trophic interactions in ecological communities (Post, 2002b; Post et al., 2000; Schriever, 2015). Habitat degradation can alter the trophic structure of an ecosystem (Dobson et al., 2011), with a high frequency and intensity of disturbance predicted to result in shorter food chains (Menge & Sutherland, 1987). However, empirical understanding of how FCL responds to disturbance has been limited by the inability to quantify this key property of trophic ecology. Stable isotope techniques offer an opportunity to investigate changes in trophodynamics from the perspective of discrete trophic levels, while still capturing the dynamics of energy flow in the food web (Post, 2002a).

In this study, we use stable isotope analysis to investigate whether changes in prey availability due to habitat degradation affect the trophic niche of a coral reef mesopredator. The specific objectives of the study were to (1) map food web trophic structure in terms of both carbon source (δ^13^C) and trophic level (δ^15^N) on degraded and healthy reefs; (2) quantify changes in the prey fish community associated with habitat degradation; (3) use stable isotopes to determine whether coral grouper altered their diets in response to changing prey availability; (4) assess whether FCL is affected by habitat degradation; and (5) investigate how grouper populations are responding to changes in trophic structure.

## 2 Materials and Methods

### 2.1 Study site

**Figure 1:**
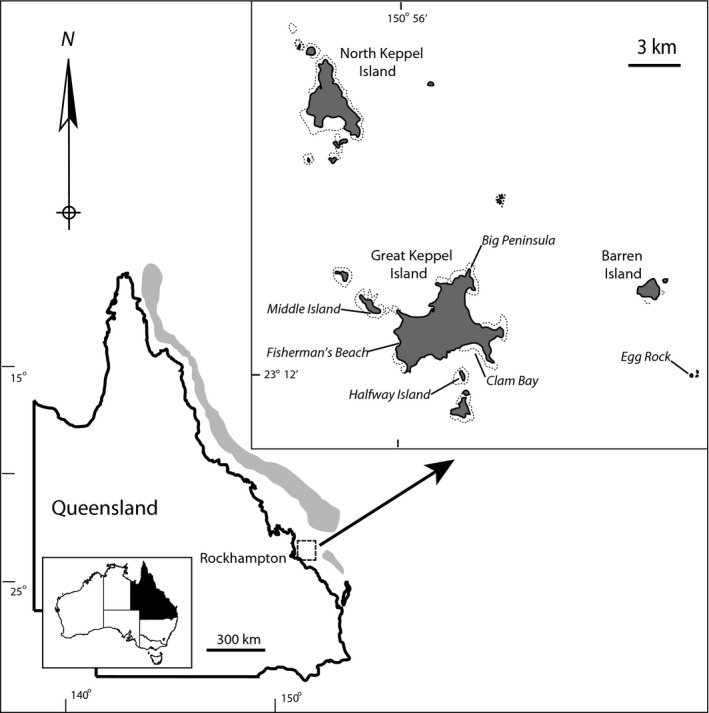
Study area in the Keppel Island Group on the Southern Great Barrier Reef, showing the approximate location of all monitoring sites where coral grouper biopsies were collected, as well as isotope food web samples

The study was carried out in the Keppel Island Group (Figure 1) in the southern section of the Great Barrier Reef, Australia. Coral reefs surrounding the Keppel Island Group were characterized by exceptionally high coral cover up until the early 2000s (Elvidge et al., 2004). A localized bleaching event in 2006 reduced coral cover by 27% (Williamson, Ceccarelli, Evans, Jones, & Russ, 2014) and, despite these reefs demonstrating the potential for fast recovery from this acute disturbance (Diaz‐Pulido et al., 2009), long‐term monitoring over the past decade has documented steady habitat degradation associated with both coral bleaching and freshwater river flood plumes (Williamson et al., 2014). A major flooding event occurred between December 2010 and January 2011 (Berkelmans, Jones, & Schaffelke, 2012), resulting in an overall decline in live hard coral cover of 37% (Williamson et al., 2014) and an increase in dead coral, rubble, and macroalgal cover (for details of site‐level changes, see Table S1). In 2009, after several years of recovery and prior to the flood plume disturbances, 75% of monitored reefs in the Keppel Islands supported at least 50% cover of live coral. In 2013, however, only 10% of monitored reefs had retained above 50% live coral cover, and 15% of reefs supported less than 5% live cover (Williamson, Ceccarelli, Rossetti, Russ, & Jones, 2016). Accompanying this habitat decline was a change in the associated prey fish community, from an assemblage characterized by large schools of plankton‐feeding damselfishes (e.g., *Chromis nitida*), to one dominated by territorial algal‐feeding damselfishes (e.g., *Pomacentrus wardi*; Williamson et al., 2014). Coral grouper (*Plectropomus maculatus*) are the dominant coral reef mesopredator in the Keppel Islands, with mean densities of 150 individuals/ha (Williamson et al., 2014).

### 2.2 Fish and benthic surveys

Reef fish and benthic communities in the Keppel Islands were surveyed prior to the flood event (2009, predisturbance), and twice after the flood event (2011 and 2013, postdisturbance). These surveys were conducted at four fringing reef sites (Clam Bay, Big Peninsula, Halfway Island, Middle Island; Figure 1) as part of a long‐term monitoring program, using underwater visual census (UVC) on SCUBA according to the methods established in Williamson et al. (2014). The fish community was quantified along 5 replicate UVC transects laid at a depth of 3–9 m parallel to the reef crest at each site. Coral grouper (*P. maculatus*) were counted in 50 × 6 m transects (300 m^2^ survey area) and assigned into 5‐cm length classes. Small‐bodied prey species (Pomacentridae and small Labridae) were counted on the return swim within a 2‐m‐wide transect (100 m^2^ survey area). The benthic composition of the sites was surveyed using a line intercept method, with benthic point samples recorded every 1 m along the 50 m transect lines.

### 2.3 Mesopredator sampling

Coral grouper dorsal muscle tissue was sourced from 36 archived biopsy probe samples collected from the five reef sites in the Keppel Islands between 2009 and 2013 (Table S2). Samples were stored in 100% high‐grade ethanol. Ethanol has been shown to have less effect on isotopic signatures than other preservatives (Ruiz‐Cooley, Garcia, & Hetherington, 2011; Sarakinos, Johnson, & Vander Zanden, 2002). However, preservation in ethanol can increase mean δ^13^C and δ^15^N values, as it acts as a fat solvent, removing isotopically light lipids from muscle samples (Carabel, Godínez‐Domínguez, Verísimo, Fernández, & Freire, 2006; Sarakinos et al., 2002; Sweeting, Polunin, & Jennings, 2004). The magnitude of effect is likely to be species specific (Kelly, Dempson, & Power, 2006) and may depend on the concentration of preservative used, the duration of preservation, and the tissue preserved (Ruiz‐Cooley et al., 2011). For a congener species (*Plectropomus leopardus*), we found that after lipid extraction, δ^13^C did not differ significantly between muscle tissue samples that had been frozen for 9 months and samples that had been stored in 100% ethanol (*t*_7.3_ = 0.03, *df* = 7.3, *p* = .98; unpublished data, see Supporting Information for details). However, to reduce any potential bias, we lipid‐extracted muscle tissue samples using chloroform–methanol (2:1) extraction prior to performing stable isotope analyses (Ruiz‐Cooley et al., 2011).

Coral grouper (*Plectropomus* spp.) undergo a marked ontogenetic diet shift once they reach approximately 20 cm standard length (SL; total length [TL] = 23.9 cm), when their diet changes from a combination of benthic invertebrates and fish to almost exclusively fish (Kingsford, 1992; St John, 1995, 1999). For this reason, we only took muscle tissue samples from coral grouper >20 cm SL (Table S2). A broad range of prey fishes have been identified in the diets of coral grouper >20 cm SL, with small‐bodied, locally abundant damselfishes (Pomacentridae) most often dominating gut content samples (Kingsford, 1992; St John, 1995, 1999; Wen, Almany, Williamson, Pratchett, & Jones, 2012). Labridae (including Scarids) and Caesionidae are also common in coral grouper diets, as well as small schooling fishes (e.g., Clupeidae and Engraulidae) when available (Kingsford, 1992; St John, 1995, 1999).

### 2.4 Food web sampling

To characterize the carbon pathways and trophic structuring at our study sites, we collected samples from lower trophic‐level fish, invertebrate species, and turf algae in May and August 2013. Samples of white muscle tissue were collected from damselfishes with different dietary niches, including the dominant algal‐feeding species (*P. wardi*), the dominant plankton‐feeding species (*C. nitida*), and two species with a mixed diet of algae and plankton (*Pomacentrus australis* and *Pomacentrus moluccensis*). A coral grouper congener (*P. leopardus*) is reported to opportunistically prey upon pelagic schools of baitfish (Kingsford, 1992), so we also sampled hardyhead silversides (*Atherinomorus* lacunosus*)* in order to account for this potential pelagic isotopic signal. Finally, to provide a baseline for the interpretation of isotope results, we collected samples of benthic turf algae and muscle tissue from filter‐feeding rock oysters to characterize the basal isotopic signatures of benthic versus pelagic carbon sources in the food web at each site.

Prey fishes were collected by SCUBA divers using handspears. All sampled fishes were euthanized using a concentrated clove oil in seawater emulsion and immediately stored on ice to preserve tissues. Tissue samples of approximately 0.5 cm^3^ were cut from the white muscle tissue between the dorsal fin and the lateral line of all fish, taking care not to include any bone or skin tissue. Turf algae samples were collected from the blades of macro algae (*Lobophora variegata*). For oysters, all connective tissue was removed and samples were taken from the main adductor muscle tissue, ensuring that samples did not contain any calcareous shell. All samples were thoroughly rinsed with distilled water, and muscle tissue samples were soaked in distilled water for 5 min, to remove salt that could cause problems with the mass spectrometry equipment. Samples were frozen in 1.5‐ml plastic vials and freeze‐dried at −50°C, 0.16 mBar for 24 hr. Dried samples were subsequently ground to a fine homogenous powder in preparation for analyses.

### 2.5 Stable isotope analysis

Bulk stable isotope analysis of carbon and nitrogen for all samples was carried out at the Great Lakes Institute for Environmental Research Laboratory at the University of Windsor, Canada. Lipids were extracted using chloroform–methanol extraction for all muscle tissue samples to ensure that differences in the fat content between species did not confound the δ^13^C results (Post et al., 2007). Algal samples were acid washed with hydrochloric acid to dissolve any calcareous matter from corals or sediment that may have contaminated the samples. Isotope ratios were calculated from 400 to 600 μg of each sample added to tin capsules and analyzed with a continuous flow isotope ratio mass spectrometer (Finnigan MAT Deltaplus, Thermo Finnigan, San Jose, CA, USA).

Stable isotope values for both carbon and nitrogen are expressed as delta (δ) values, equal to parts per thousand (‰) deviation from the standard, using the equation:

$$\delta X=\left[\frac{R_{\mathrm{sample}}}{R_{\mathrm{standard}}}-1\right]\times 1,000,$$

where *X* is ^13^C and *R* is the ratio ^13^C:^12^C for δ^13^C, or *X* is ^15^N and *R* is the ratio ^15^N:^14^N for δ^15^N (Peterson & Fry, 1987). The standard reference material used for carbon and nitrogen were Pee Dee Belemnite carbonate and atmospheric nitrogen, respectively. FCL can be approximated by assessing the TP at which high‐level consumers in the ecosystem are feeding. TP of all consumers was calculated from the δ^15^N stable isotope results according to the equation below (Hussey et al., 2014). The mean δ^15^N signature of all filter‐feeding oysters sampled was used as the trophic level 2 baseline, with a mean TP = 2, from which to calculate the relative positions of all other groups.

$$\text{TP}=-\log\left(\frac{\log\left(\delta^{15}N_{\lim}-\delta^{15}N_{\mathrm{base}}\right)-\log\left(\delta^{15}N_{\lim}-\delta^{15}N_{\mathrm{TP}}\right)}{k}\right)+{\text{TP}}_{\mathrm{base}},$$

where δ^15^N_base_ is the isotope value for a known baseline consumer in the food web, *k* is the rate at which δ^15^N_TP_ approaches δ^15^N_lim_ per TP step.

And, estimates of *k* and $\delta^{15}N_{\lim}$ are given by:

$$k=-\log\left(\frac{\beta_{0}-\delta^{15}N_{\lim}}{-\delta^{15}N_{\lim}}\right),$$

$$\delta^{15}N_{\lim}=\left(\frac{\beta_{0}}{-\beta_{1}}\right).$$

with intercept, β_0_ = 5.924 and slope β_1_ = −0.271 characterizing the change in δ^15^N as dietary δ^15^N values increase, given by the meta‐analysis in Hussey et al. (2014).

### 2.6 Data analyses

To characterize trophic structure within the food web in terms of both carbon sources and trophic levels, the isotopic signatures of all samples were plotted in isotopic space using a δ^13^C by TP biplot. For all subsequent analyses, grouper sampled from Egg Rock were excluded due to the lack of data on prey fish and benthic communities from this offshore site.

To investigate the source of variability in the δ^13^C and δ^15^N signal among coral grouper tissue samples, we constructed a set of hierarchical (mixed effects) linear models. Covariates of interest included percentage live hard coral cover (as a measure of habitat condition), abundance of planktivorous pomacentrids (the prey fish which dominate on reefs with higher coral cover; Table S3), abundance of territorial benthic‐feeding pomacentrids (prey fish which dominate on more degraded reefs with lower coral cover; Table S3), and a ratio of the logged abundances of planktivorous pomacentrids to territorial pomacentrids (to examine the effect of their relative dominance on a reef). To ensure that any changes in isotopic signature were not simply due to ontogenetic diet shifts, TL of individual fish was included as a covariate. Location can also be an important driver of isotope ratios, particularly in an inshore system that is under strong terrestrial influence from river outflow and flooding events, we included a covariate for distance from shore, measured as the straight‐line distance from the middle of each site to the high water mark due west on the mainland. Model variables extracted from long‐term monitoring data were averaged over the 2–3 monitoring sites closest to the location and date at which each grouper tissue sample was taken. A random factor was also included for site and year to account for any unexplained variance in the data.

Rather than relying on arbitrary methods for model selection, we calculated a model‐averaged estimate for each standardized variable across all models using multimodel inference (Burnham & Anderson, 2002) to estimate a weighted average of parameter estimates based on model uncertainty (Akaike weights) of all models.

Finally, we looked at how coral grouper abundance changed over the study period, again using a hierarchical linear (mixed effects) model, to test what factors may be responsible for driving any observed changes, including the same covariates tested in the previous isotope model, with the exception of TL. Total available prey biomass was added as an additional covariate to account for the different body sizes of prey species, which would not be captured in abundance data alone. Based on what is known about the diet of the congener, *P. leopardus* (Kingsford, 1992), we included all species from the families Labridae (including juvenile parrotfishes) and Pomacentridae, with a maximum TL of 20 cm or less (Froese & Pauly, 2015), and for which at least three individuals had been recorded in the fish community monitoring dataset. Individual biomass estimates for each species were calculated according to the equation *W = aLb* where *W* is the weight, *L* is maximum TL for the species, and *a* and *b* are species‐specific volumetric constants sourced from FishBase (Froese & Pauly, 2015). This was then multiplied by the total number of individuals recorded for any given species, site, and year and summed to provide an estimate of the total biomass available to mesopredators.

All data exploration was carried out in R following the protocol described in Zuur, Ieno, and Elphick (2010). Cleveland dotplots were used to inspect the variables for outliers. Pairplots and variance inflation factors values were used to assess colinearity, and multipanel scatterplots were used to visualize relationships. Model selection was based on the Akaike information criterion.

## 3 Results

### 3.1 Shift in prey fish availability

**Figure 2:**
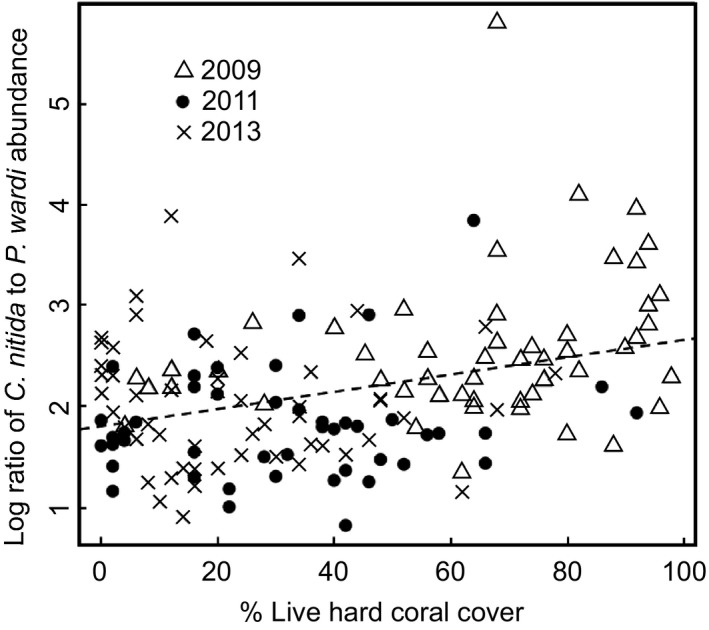
Relationship between percentage live hard coral cover and ratio of the dominant prey fish species available to mesopredators in the fish community. At higher percentage live hard coral cover, planktivorous damselfishes (*Chromis nitida*) dominate the available prey fish community, while at low coral cover, territorial benthic‐feeding species (*Pomacentrus wardi*) are relatively more abundant

Loss of live hard coral cover with habitat degradation in the Keppel Islands between 2009 and 2013 (Table S1) was associated with a change in the dominant prey fish species (Figure 2), from planktivorous damselfishes (*C. nitida*) to territorial benthic‐feeding damselfishes (*P. wardi*; 0.008 [0.004, 0.012]; maximum‐likelihood estimate [95% confidence interval]). This likely represents a shift in the principal carbon source available to piscivores such as coral grouper.

### 3.2 Community trophic structure

**Figure 3:**
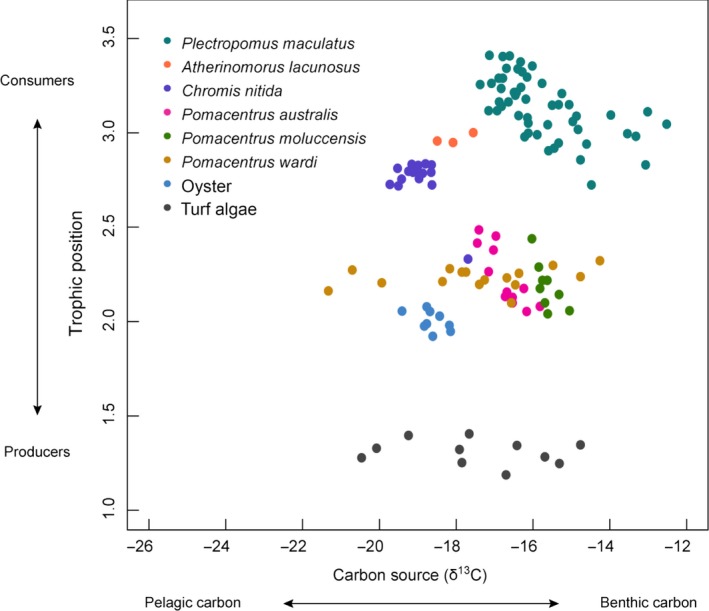
Isotope biplot showing the trophic structuring within the Keppel Islands' coral reef food web in terms of carbon source (δ^13^C) and trophic position, which is a function of δ^15^N. Sampling sites are indicated by different shaped symbols, and species are designated by color

Stable isotopes identified distinct trophic structuring within the Keppel Islands' coral reef food web (Figure 3) associated with distinct carbon pathways (δ^13^C). Oysters, which as filter feeders, were considered to characterize the pelagic carbon signal in the system, had a mean δ^13^C value of −18.19‰ (±0.10 SE), a signal that was tracked by that of *C. nitida*, the pelagic plankton‐feeding damselfish (−18.69‰, ±0.07). The benthic basal carbon signal of the algae was less negative and considerably more variable (−17.21‰, ±0.43) than the planktonic signal. As a producer, the TP of turf algae should be 1, but our results show an inflated TP for these samples (1.31 ± 0.02). This variability in both δ^13^C and TP (calculated from δ^15^N) could be due to contamination of algal samples from other carbon sources from reef and land‐based sediment and detritus. This benthic basal carbon signal was reflected in the isotopic signatures of benthic algal‐feeding damselfish *P. wardi* (−16.88‰, ±0.35). The TP values for all other groups sampled concurred with what is known about the ecological niches of these species (Figure 3).

### 3.3 Variation in coral grouper δ^13^C

**Figure 4:**
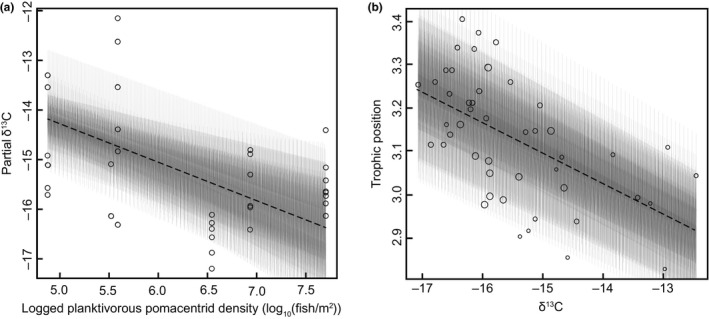
(a) The marginal change in δ^13^C signature of coral grouper (*Plectropomus maculatus*) sampled from the Keppel Islands between 2009 and 2013 was best explained by the decrease in planktivorous prey species in the fish community. (b) Relationship between δ^13^C (carbon source) and trophic position (calculated from δ^15^N) in coral grouper

There was a substantial amount of variability in isotopic signals among the coral grouper sampled, with δ^13^C values ranging from −17.07‰ to −12.46‰ (Figure 3). The abundance of planktivorous damselfishes was the strongest driver of this variation in coral grouper δ^13^C values (−0.69 [−0.79, −0.58]; see Table S4 standardized parameter estimates). Grouper sampled at sites dominated by planktivorous damselfishes had a more negative (pelagic) δ^13^C signal, while fish from more degraded sites, where territorial damselfishes are more dominant, had a less negative (benthic) δ^13^C signal in their muscle tissue (Figure 4a), with an overall enrichment toward a benthic signature in grouper δ^13^C over time (Fig. S1).

Percentage live hard coral cover was highly correlated with the abundance of planktivorous pomacentrids (Pearson's correlation coefficient; *r* = .8), as were the abundance of territorial pomacentrids (*r* = −.7), and the ratio of the abundances of planktivorous pomacentrids to territorial pomacentrids (*r* = .7). These covariates were therefore excluded from the final model. Fish size (TL) was a poor predictor of grouper δ^13^C signature (0.00 [−0.01, 0.01]), as was distance from shore (−0.04 [−0.11, 0.04]), indicating that, among the fish sampled for this study, there was no confounding effect of either individual size or terrestrial influence on δ^13^C.

### 3.4 Variation in coral grouper δ^15^N

The isotopic signature of coral grouper (*P. maculatus*) placed them at the highest TP of the species sampled (TP = 3.13 ± 0.02 SE), as would be expected for a reef mesopredator, but there was also a great deal of variation in TP between individuals, with values ranging from 2.72 to 3.41. The fish with the highest TPs were also those with the most negative (pelagic) δ^13^C signals (Figure 4b). This indicates that coral grouper that were feeding predominantly on planktivorous prey species were also on reefs with greater trophic complexity and longer food chains.

Akaike information criterion‐based model averaging of hierarchical linear mixed effects models showed the two strongest predictors of δ^15^N to be the abundance of territorial pomacentrids (0.396 [−0.17, 0.96]), and grouper TL (0.013 [0.001, −0.03]).

### 3.5 Coral grouper population response

**Figure 5:**
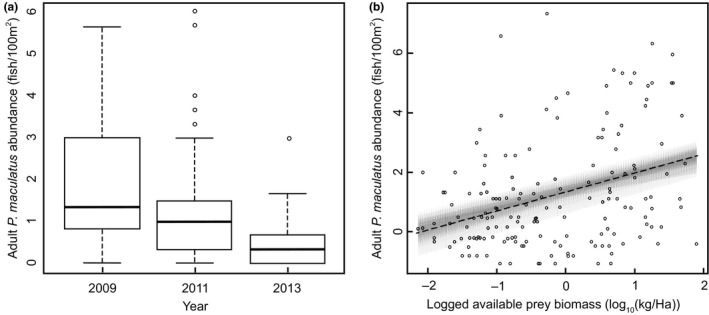
(a) Mean abundance of adult (SL > 20 cm) coral grouper (*Plectropomus maculatus*) on the reefs of the Keppel Island Group in (*n* = 165). The dark line indicates the median of the data, boxes represent the bounds of the first and third quartiles, with whiskers extending to 1.5 times the interquartile range past these points. (b) Relationship of adult coral grouper abundance to the total logged biomass (log(kg/Ha^1^)) of prey fishes available to them in the reef fish community

Adult coral grouper (*P. maculatus*, SL > 20 cm) abundance decreased steadily with time over the years examined in this study (Figure 5a), from a mean (±SE) of 2.077 ± 0.209 fish/100 m^2^ in 2009, to 1.170 ± 0.192 in 2011, and 0.449 ± 0.082 in 2013. The total prey biomass available to mesopredators also diminished over the course of the study (Fig. S2) and was the most important factor associated with the grouper population decline (0.634 [0.480, 0.788]; Figure 5b). The abundance of planktivorous pomacentrids was strongly correlated with total available prey biomass (Pearson's correlation coefficient = .7). Percentage live hard coral cover (0.017 [0.010, 0.024]), the abundance of territorial pomacentrids (0.0254 [0.013, 0.038]), and distance from shore (0.029 [−0.145, 0.202]) were all much weaker predictors of coral grouper abundance.

## 4 Discussion

Fluctuations in prey availability linked to environmental change are becoming increasingly common, and a predator's dietary plasticity may be critical to their persistence in a given ecosystem (Berumen, Pratchett, & McCormick, 2005; Peers, Wehtje, Thornton, & Murray, 2014). Increased knowledge of how coral reef mesopredators respond to changing prey availability is therefore key to predicting how coral reef trophic dynamics will be affected by widespread habitat degradation. This study presents some of the first empirical evidence that piscivorous mesopredators may be able to adapt their diets in response to such changes.

Prey switching often occurs when the relative abundance of prey species is altered and predators modify their diets to exploit this change in available resources (Berumen et al., 2005). In the present study, differences in the δ^13^C signature of coral grouper in the Keppel Island Group suggest that they are capable of adapting their diets in response to changes in prey availability due to habitat degradation. While this may appear to be an effective strategy, costs associated with shifts in diet may not be evident in the short term. Previous studies documenting prey switching in marine and terrestrial species have linked facultative dietary shifts to detrimental effects (e.g., Cohen et al., 2014; McNamara & Lonsdale, 2014; Pratchett, Wilson, Berumen, & McCormick, 2004). For example, when switching to less preferred prey species, there may be a loss of condition due to reduced nutritional quality (Berumen et al., 2005; Pratchett et al., 2004), or due to increased energetic costs involved in capturing alternative prey (Cohen et al., 2014). Such sublethal effects are not immediately evident in the population and may result in reduced fecundity (Jones & McCormick, 2002), growth rates (Feary, McCormick, & Jones, 2009; Kokita & Nakazono, 2001), or delayed age of maturity (Jonsson, Jonsson, & Finstad, 2013); all of which can have a significant impact on the population in the longer term (Graham et al., 2007). Dietary adaptability may therefore only ameliorate the effects of habitat degradation in the short term. If a consumer's habitat recovers rapidly following disturbance, then prey switching could be an effective way for longer‐lived mesopredators to survive until the system recovers, despite loss of condition in the short term.

Shortening of food chains is often symptomatic of deterioration of ecosystem function, frequently driven by a loss of top consumers in an ecosystem (Estes et al., 2011). Dobson et al. (2011) also highlight an important process called trophic downgrading, whereby a thinning of the food web due to a loss of species diversity results in a decrease in the mean trophic level of consumers. Such changes in FCL therefore have the potential to influence key ecological dynamics such as rates of primary productivity, nutrient cycling, and carbon flow (Oksanen & Oksanen, 2000; Pace, Cole, Carpenter, & Kitchell, 1999; Persson, 1999). With the loss of live coral cover in the Keppel Islands followed by a decline in fish diversity (Williamson et al., 2014), we document that a species of mesopredator (coral grouper, *P. maculatus*) shows variation in its TP, indicating differences in FCL, associated with an increase in territorial benthic pomacentrids. These effects were matched by those of increasing body size, a well‐known driver of increasing isotope‐derived TL among fishes (Jennings, Pinnegar, Polunin, & Boon, 2001).

Grouper feeding at lower trophic levels also had a more enriched δ^13^C signature, indicative of a diet rich in benthic herbivorous species. These species (e.g., *P. wardi*) were found to be dominant on reefs with decreased live coral cover and low fish species diversity (Williamson et al., 2014), suggesting a thinning of the food web on the degrading Keppel Island reefs. According to Dobson et al. (2011), a subsequent stage is a rapid shortening of the food chain as trophic levels are lost from top to bottom, leading to a simplification of the food web. Coral grouper populations in the Keppel Islands are already in decline, a trend that appears to be primarily related to a decrease in the total available biomass of prey, supporting the suggestion by Williamson et al. (2014) that the reduction in prey fish abundance is largely responsible for the decreased abundance of mesopredators.

While disturbance has already impacted the reef fish community in the Keppel Islands considerably, the habitat degradation on these reefs is relatively recent, with the majority of live coral loss occurring since 2011 (Diaz‐Pulido et al., 2009; Williamson et al., 2014). It is therefore possible that these effects may become more pronounced in the longer term (Graham et al., 2007) as intact skeletons from dead coral that were maintaining a degree of structural complexity on the Keppel reefs will be lost as these reefs degrade further. Structural complexity is crucial in not only supporting coral reef fish communities (Graham & Nash, 2013; Nash, Graham, Wilson, & Bellwood, 2013; Syms & Jones, 2000), but also in facilitating successful predation by mesopredators such as coral grouper that rely on shelter for ambush predation (Kerry & Bellwood, 2012). Mesopredators may therefore decline further as their prey base is increasingly altered, and predation becomes more challenging, requiring greater energy investment.

The effects of widespread habitat degradation on long‐lived reef mesopredators remain poorly understood, as sublethal effects may not be apparent in the short term. These species are often of great economic and social value (Cinner et al., 2009; GBRMPA, 2014) and play a key functional role in the trophodynamics of coral reef ecosystems, transferring energy up the food chain (Polovina, 1984), and potentially offering a stabilizing effect in postdisturbance communities (Loeuille, 2010; McCann, Hastings, & Huxel, 1998). Improving our understanding of how habitat degradation impacts this functional group, particularly at a sublethal level, is therefore a high priority for future research.

This study provides evidence that the trophodynamics of mesopredators could become altered due to habitat degradation and altered prey availability. Our results also illustrate the utility of stable isotope analyses in detecting the early stages of trophic downgrading in a marine ecosystem. We conclude that while the ability of mesopredators to modify their diets may be effective at ameliorating the effects of habitat degradation on coral grouper in the short term, altered trophic structure, decreased total prey availability, and sublethal effects may have detrimental consequences for mesopredator populations in the longer term. This study contributes to improving forecasts about how coral reef ecosystems will respond to habitat degradation and environmental change in the future, facilitating better‐informed management decision‐making, particularly with respect to coral reef fisheries.

## Conflict of Interest

None declared.
