# Supplementary material for: Coral reef mesopredators switch prey, shortening food chains, in response to habitat degradation
Source: Ecol Evol. 2017 Mar 18;7(8):2626–35. doi: 10.1002/ece3.2805 (PMC5395445; doi:10.1002/ece3.2805)
Supplement: Supplementary file 1 [file ECE3-7-2626-s001.docx]

**Coral reef mesopredators switch prey, shortening food chains, in response to habitat degradation**

Tessa N. Hempson, Nicholas A. J. Graham, M. Aaron MacNeil, David H. Williamson,

Geoffrey P. Jones and Glenn R. Almany

**Supporting Information**

**Table S1.** Composition of the benthic cover of reefs in the inner Keppel Island Group on the southern Great Barrier reef in 2009, 2011 and 2013, showing mean percentage cover (±standard error; SE) of the three dominant cover types and mean (±SE) structural complexity index (SCI; slope x rugosity).

|  |  |  |  | Live hard coral cover (%) | |  | Macroalgae cover (%) | |  | Dead coral & rubble cover (%) | |  | SCI | |
| --- | --- | --- | --- | --- | --- | --- | --- | --- | --- | --- | --- | --- | --- | --- |
| Site | Year | n |  | Mean | ± SE |  | Mean | ± SE |  | Mean | ± SE |  | Mean | ± SE |
|  |  |  |  |  |  |  |  |  |  |  |  |  |  |  |
| Big Peninsula | 2009 | 10 |  | 81.60 | 3.04 |  | 0.00 | 0.00 |  | 10.00 | 1.58 |  | 9.80 | 0.82 |
|  | 2011 | 10 |  | 49.40 | 7.88 |  | 0.00 | 0.00 |  | 31.00 | 6.42 |  | 7.36 | 0.65 |
|  | 2013 | 10 |  | 32.20 | 5.62 |  | 0.20 | 0.20 |  | 27.20 | 5.58 |  | 5.92 | 0.41 |
|  |  |  |  |  |  |  |  |  |  |  |  |  |  |  |
| Clam Bay | 2009 | 15 |  | 46.67 | 8.37 |  | 40.53 | 10.19 |  | 7.20 | 1.55 |  | 5.73 | 0.14 |
|  | 2011 | 15 |  | 33.60 | 7.34 |  | 0.27 | 0.18 |  | 60.93 | 7.85 |  | 4.54 | 0.31 |
|  | 2013 | 15 |  | 20.80 | 6.51 |  | 17.87 | 3.59 |  | 51.87 | 4.39 |  | 4.48 | 0.25 |
|  |  |  |  |  |  |  |  |  |  |  |  |  |  |  |
| Halfway Island | 2009 | 15 |  | 68.00 | 6.11 |  | 18.67 | 7.08 |  | 8.40 | 2.34 |  | 6.29 | 0.39 |
|  | 2011 | 15 |  | 36.27 | 4.14 |  | 0.00 | 0.00 |  | 51.73 | 5.28 |  | 7.13 | 0.41 |
|  | 2013 | 15 |  | 21.87 | 3.57 |  | 3.60 | 1.05 |  | 62.53 | 4.57 |  | 4.37 | 0.17 |
|  |  |  |  |  |  |  |  |  |  |  |  |  |  |  |
| Middle Island | 2009 | 15 |  | 67.60 | 4.73 |  | 15.73 | 4.76 |  | 15.07 | 2.77 |  | 7.50 | 0.19 |
|  | 2011 | 15 |  | 26.67 | 5.17 |  | 0.00 | 0.00 |  | 66.53 | 7.08 |  | 4.73 | 0.23 |
|  | 2013 | 15 |  | 17.33 | 5.11 |  | 24.80 | 5.51 |  | 52.13 | 6.61 |  | 4.39 | 0.32 |
|  |  |  |  |  |  |  |  |  |  |  |  |  |  |  |

**Table S2.** Number (n) of coral grouper (*Plectropomus maculatus*) samples collected at five sites, during three sampling periods in the Keppel Island Group on the Great Barrier Reef, showing mean total length (TL, cm) ± standard error (SE) for each site and year sampled.

|  |  | Big Peninsula | | |  | Clam Bay | | |  | Egg Rock | | |  | Halfway Island | | |  | Middle Island | | |  |  |
| --- | --- | --- | --- | --- | --- | --- | --- | --- | --- | --- | --- | --- | --- | --- | --- | --- | --- | --- | --- | --- | --- | --- |
| Year |  | Mean TL  (cm) | ±SE | n |  | Mean TL  (cm) | ±SE | n |  | Mean TL  (cm) | ±SE | n |  | Mean TL  (cm) | ±SE | n |  | Mean TL  (cm) | ±SE | n |  | **Total** |
| 2009 |  |  |  | 0 |  | 41.7 | 1.2 | 9 |  |  |  | 0 |  |  |  | 0 |  |  |  | 0 |  | 9 |
| 2012 |  | 60.5 | 4.5 | 2 |  | 53.2 | 3.2 | 7 |  | 59.3 | 3.4 | 6 |  | 52.5 | 4.4 | 6 |  | 45.0 | 1.2 | 6 |  | 27 |
| 2013 |  |  |  | 0 |  |  |  | 0 |  | 51.7 | 4.6 | 6 |  | 25.1 | 0.6 | 6 |  |  |  | 0 |  | 12 |
| Total |  |  |  | 2 |  |  |  | 16 |  |  |  | 12 |  |  |  | 12 |  |  |  | 6 |  | 48 |

**Table S3.** Prey fish species functional group allocations

| Family | Species | Functional Group |
| --- | --- | --- |
| Pomacentridae | *Chromis amboinensis* | Planktivorous pomacentrid |
|  | *Chromis atripectoralis* | Planktivorous pomacentrid |
|  | *Chromis nitida* | Planktivorous pomacentrid |
|  | *Chromis ternatensis* | Planktivorous pomacentrid |
|  | *Pomacentrus lepidogenis* | Planktivorous pomacentrid |
|  |  |  |
|  | *Hemiglyphidodon plagiometapon plagiometapon* | Territorial pomacentrid |
|  | *Neoglyphidodon melas* | Territorial pomacentrid |
|  | *Pomacentrus bankanensis* | Territorial pomacentrid |
|  | *Pomacentrus wardi* | Territorial pomacentrid |
|  | *Stegastes apicalis* | Territorial pomacentrid |
|  | *Stegastes fasciolatus* | Territorial pomacentrid |
|  |  |  |

**Table S4.**  Standardised AIC-weighted model-averaged parameter estimates with 95% confidence intervals for models of stable isotope (δ^13^C and δ^15^N) signatures in coral grouper (*Plectropomus maculatus*).

| Response variable | Model parameter | Estimate | lower 95% limit | upper 95% limit |
| --- | --- | --- | --- | --- |
|  |  |  |  |  |
| δ^13^C | Distance from shore | -0.38 | -0.98 | 0.22 |
|  | Total length | 0.02 | -0.66 | 0.69 |
|  | Planktivorous pomacentrid abundance | -1.30 | -2.01 | -0.59 |
|  |  |  |  |  |
| δ^15^N | Total length | 0.60 | 0.35 | 0.86 |
|  | Territorial pomacentrid abundance | 0.57 | 0.38 | 0.75 |
|  |  |  |  |  |


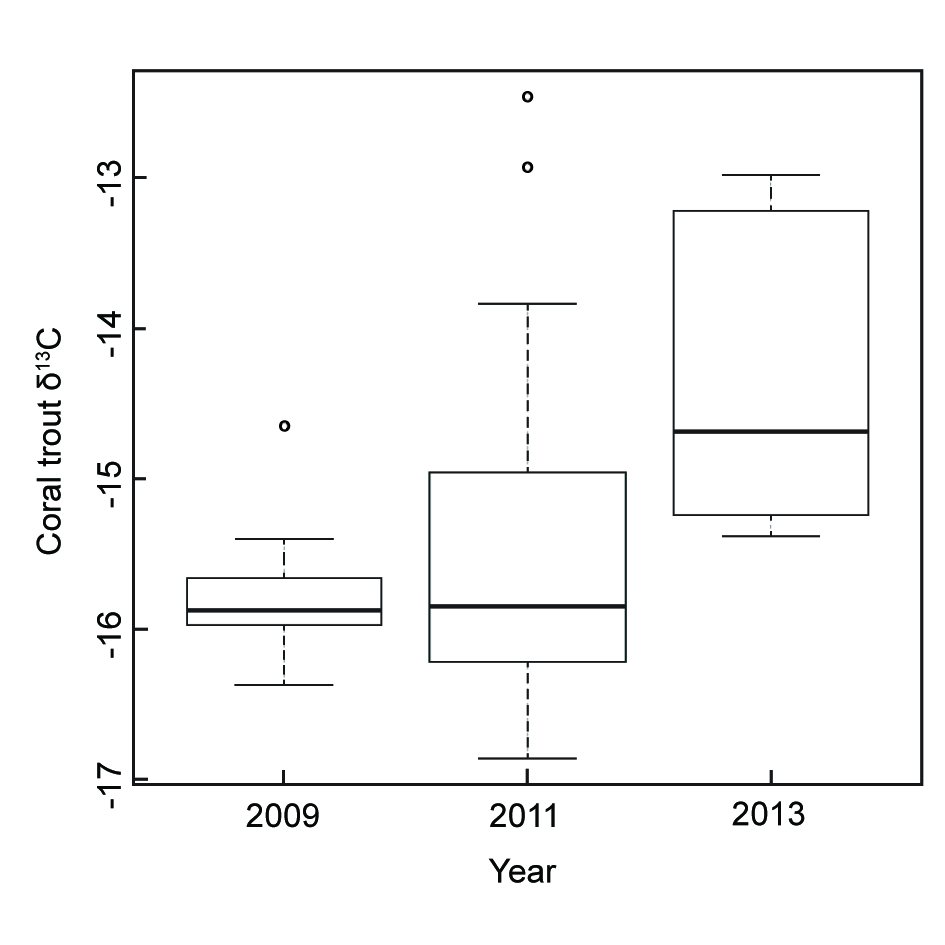


**Fig. S1** Shift in the δ^13^C signal in coral grouper (*Plectropomus maculatus)* between 2009 (n=9), 2011 (n=27), and 2013 (n=12), from a more negative (i.e. planktonic) signal, to more positive (i.e. benthic) signal. The dark line indicates the median of the data, boxes represent the bounds of the first and third quartile, with whiskers extending to 1.5 times the interquartile range past these points.

**
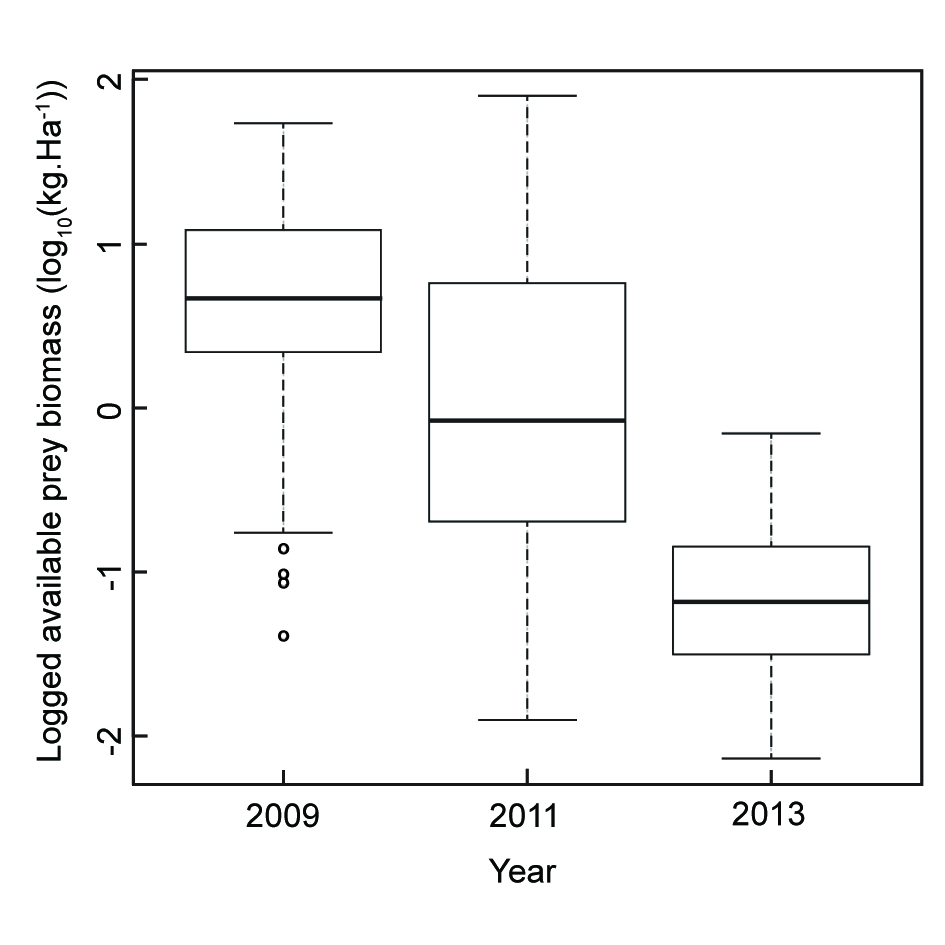
**

**Fig. S2** Change in logged total prey biomass available to coral grouper in the Keppel Islands between 2009 (n=9), 2011 (n=27), and 2013 (n=12). The dark line indicates the median of the data, boxes represent the bounds of the first and third quartile, with whiskers extending to 1.5 times the interquartile range past these points.

**Methods of Ethanol Storage Experiment**

In November 2014, 20 clean white muscle tissue samples of 0.5 cm^3^ were taken from a single fresh adult specimen of *Plectropomus leopardus*. Tissue samples were thoroughly rinsed with distilled water to remove any salt or other contaminants. Ten samples were stored in 100% ethanol, while the other 10 samples were frozen in sealed vials. After 9 months of storage, the samples stored in ethanol were again rinsed in distilled water to remove the preservative, and all samples were freeze-dried, lipid extracted and analysed for δ^13^C bulk stable isotope content.
